# Study protocol for a randomised controlled trial of exercise training in pulmonary hypertension (ExTra_PH)

**DOI:** 10.1186/s12890-018-0586-8

**Published:** 2018-03-01

**Authors:** Norman R. Morris, Menaka Louis, Wendy Strugnell, Julie Harris, Aaron Lin, John Feenstra, Helen Seale

**Affiliations:** 1 Allied Health Collaborative, The Prince Charles Hospital, Brisbane, Australia; 2 School of Allied Health Sciences, Griffith University, Gold Coast, QLD Australia; 3 Menzies Health Institute, Queensland and Griffith University, Gold Coast, Australia; 4 Queensland Lung Transplant Service, The Prince Charles Hospital, Brisbane, Australia; 5 Richard Slaughter Centre for Medical Excellence in Cardiovascular Magnetic Resonance Imaging, The Prince Charles Hospital, Brisbane, Australia

**Keywords:** Pulmonary hypertension, Exercise training, Randomised controlled trial, Exercise cardiac magnetic resonance imaging, Right heart function

## Abstract

**Background:**

Exercise training is an integral component of evidence-based management programs for many chronic cardiac and respiratory conditions. Despite this, there are limited high-quality studies available on the significance of exercise training in pulmonary hypertension (PH). The aim of this study is to evaluate the short and long-term effectiveness of exercise training in PH patients in terms of exercise capacity, quality of life, cardiac function and disease progression.

**Methods:**

This randomized control trial will aim to recruit 50 medically stable PH patients categorised as New York Heart Association functional classification II-III. Participants will be randomly allocated to either the supervised exercise training group or usual care group for the 8-week study period. Exercise training will be conducted in an outpatient setting. Measurements at baseline and following the 8-week study period include exercise capacity (6 min walk distance and cardiopulmonary exercise test), cardiac function (exercise cardiac magnetic resonance imaging [CMRI] and echocardiography), health-related quality of life (Cambridge Pulmonary Hypertension Outcome Review), adverse responses to exercise training and time to clinical worsening. In addition, participants will be followed up for a minimum of 2 year period from commencement of the study so as to monitor long-term clinical outcomes i.e. time to clinical worsening.

**Discussion:**

This study will determine whether an 8-week outpatient based supervised exercise training program is safe and beneficial for medically stable PH patients in the short and long term. This will be the first study to examine the impact of exercise training on right heart function using exercise CMRI. Results from the study will contribute new knowledge in relation to the impact of exercise training on cardiac function, long-term prognosis and inform clinical practice guidelines for this patient population. Moreover, the study will add to our understanding regarding the efficacy of exercise training in individuals with PH in an outpatient setting.

**Trial registration:**

Australia and New Zealand Clinical Trials Registry: ACTRN12616001467426. Registered 21st October, 2016.

## Background

Pulmonary hypertension (PH) is a complex condition that is defined as an elevation in mean pulmonary artery pressure of greater than 25 mmHg at rest [1] and pulmonary capillary wedge pressure less than 15 mmHg. It has multiple pathogenetic mechanisms and is typically a progressive disorder that can lead to right heart failure and premature death [2]. Whilst recent advances in medical therapy for PH have extended the time to clinical worsening, many patients continue to experience exertional symptoms of dyspnoea and fatigue which, in turn, result in a reduction in functional capacity and quality of life (QoL) [3].

Given the reduced functional capacity in PH, it would appear logical to extrapolate the results of exercise training studies in other chronic cardiac and respiratory conditions to the PH patient population. In other chronic conditions, exercise training has well established safety and efficacy in terms of improving symptoms, exercise capacity and QoL, and thus is recognised as a fundamental management strategy [4]. Despite this, exercise training has not routinely been encouraged for patients with PH due to the concern that it may contribute to disease progression and an increased risk of serious adverse events [5, 6].

Recent studies have challenged these assumptions regarding the risks of exercise training in PH and suggested exercise can be both beneficial and safe, even for patients with severe disease [7, 8]. Indeed, recently published systematic reviews demonstrated improved exercise capacity and quality of life following exercise training in PH [9–12]. Based on the results of these studies, updated international guidelines now tentatively recommend (strength of recommendation, IIb) supervised exercise training in PH [13]. However, it has been recognised amongst PH clinical experts and the guideline authors themselves that current evidence is limited and that additional high quality studies are required to establish the efficacy and long-term effects of exercise training in PH [5, 13]. Limitations include a paucity of high quality randomised controlled trials (RCTs) and the fact that many of the studies have used a highly supervised exercise training conducted in an inpatient setting [13, 14]. Moreover, the authors recognise that the mechanisms for improvement following exercise training remain unknown as are the effects on long term outcomes [13].

Currently, the evidence base of high quality studies examining exercise training remains narrow [15]. To date, the results of only 4 small RCT’s have been published [8, 16–18]. Of these, three have been published by the same group of authors [8, 17, 18] using a supervised, 3-weeks inpatient exercise program. Although the training adaptations were more favourable during the intensive inpatient training period compared with the subsequent home program, the results of this expensive and impractical exercise training paradigm are difficult to translate to the outpatient rehabilitation models that are widely adopted in Australia and around the world. As a result, to date only one small RCT (*n* = 20) has been published by Chan et al. [16] which examined the impact of exercise training in an outpatient based environment.

Whilst there is preliminary evidence that exercise training improves skeletal muscle oxidative capacity in PH [19], there have been no studies on the effect of exercise training on right ventricular function, overall cardiac function and long-term prognosis. Further robust studies are therefore necessary to assess the efficacy and safety of outpatient-based exercise training programs and to determine the impact of exercise training on cardiac function and prognostic outcomes. As such, the objectives of the present study are to examine the safety and impact of outpatient-based exercise training on cardiac function, and short- and long-term clinical outcomes in medically stable patients with PH. We hypothesise that supervised outpatient-based exercise training is safe for the PH patient population and will result in clinically significant benefits in terms of a reduction in disease severity and delayed time to clinical worsening, as well as improved exercise capacity, QoL and cardiac function.

## Methods

### Study design

**Fig. 1:**
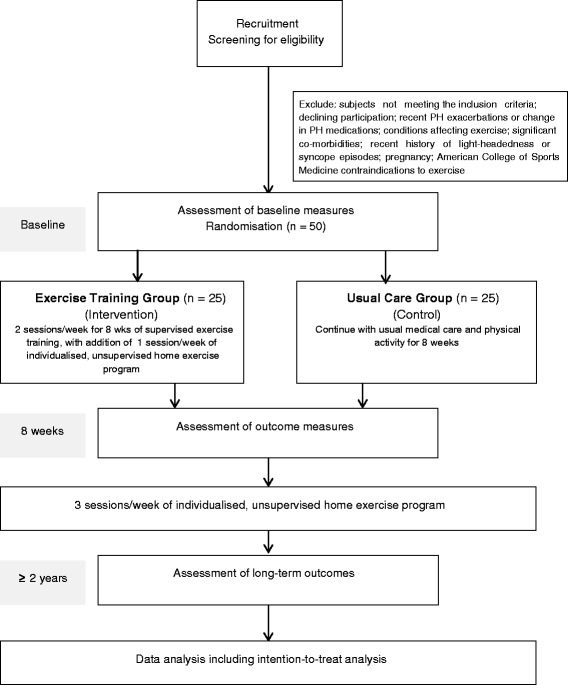
Study flow chart

This study will be a randomised controlled trial using concealed allocation and blinded outcome assessors and data analysts. Figure 1 illustrates the flow of the study from recruitment and screening for eligible participants to follow-up outcome assessments and data analysis.

### Participants

Participants will be recruited over a 3 year period from the cohort of PH patients under the care of the Queensland Centre for Pulmonary Transplantation and Vascular Disease Unit at The Prince Charles Hospital, Brisbane, Queensland. Strategies for improving participant recruitment will include regular meetings with the PH clinical team and liaising with the primary PH physician (JF) and the PH assessment physiotherapist. Inclusion criteria include: age over 18 years, a confirmed diagnosis of PH based on the results of a right heart catheterization procedure (mean pulmonary artery pressure > 25 mmHg and pulmonary capillary wedge pressure < 15 mmHg) and New York Heart Association functional classification II-III. Eligible participants also will be on stable PH medical therapies and have had no exacerbations in the previous 3 months.

Participants will be excluded if they have had a change in their PH medication regime in the last 3 months or have significant co-morbidities including left-sided heart failure. Other exclusion criteria include pregnancy, recent history of light-headedness during exercise or syncope episodes in the last month and significant musculoskeletal and neurological conditions likely to adversely impact exercise performance. Patients will also be excluded if they have any of the contraindications to exercise as per the American College of Sports Medicine (ACSM) guidelines [20].

Eligible participants will be contacted by a member of the research team and provided with verbal and written information regarding the study. Informed written consent will be obtained prior to participation in the study. The study has been approved by the Metro North Hospital and Health Service - The Prince Charles Hospital Human Research Ethics Committee (HREC). Changes to the protocol will be approved by the HREC. The trial is registered with the Australia and New Zealand Clinical Trials Registry: ACTRN12616001467426.

### Randomisation

Eligible participants will be randomised into parallel groups with a 1:1 allocation ratio to either the exercise training group or the control group after completion of the baseline measures. A concealed allocation procedure will be performed by a blinded investigator using permuted block randomisation of 10 subjects and sealed, opaque envelopes. This schedule will ensure a close balance in the number of participants assigned to each group.

### Intervention group

Participants randomly allocated to the intervention group will participate in an 8-week, twice weekly outpatient supervised exercise training program. The exercise training program will be conducted in an outpatient rehabilitation setting and supervised by an experienced physiotherapy clinician who will closely monitor the participants’ exercise performance and provide appropriate exercise progression. Details of exercise training sites can be obtained at the Clinical Trial Registry ACTRN12616001467426.

The aim is for each participant to complete 20 min each session of cycling and treadmill exercises (40 min in total). Each participant will commence training in the first session by undertaking a minimum of 10 min of cycling and 10 min of treadmill training. Interval training, consisting of 60 s of exercise and 120 s of rest, will be used if participants are unable to complete 10 min of continuous exercise. The duration of cycling and treadmill exercises will be up-titrated such that participants are completing 20 min each session of cycling and treadmill exercises by the end of the second week.

The initial treadmill speed will be set at 60–70% of six-minute walk test (6MWT) speed and titrated up to 90% of 6MWT speed as tolerated over the first 4 weeks of training, by monitoring training heart rate (HR), rating of perceived exertion (RPE) and leg fatigue on the modified Borg scale will be determined. Training intensity will be maintained at this HR and RPE for the remainder of the 8-week training period. An upper limit for exercise intensity will be set at 85% of age-predicted maximum HR and 4 on the 0–10 modified Borg RPE scale. Cycle intensity will be calculated using the 6MWT distance from standard equations [21]. The initial cycle training intensity will be prescribed at 60% of the estimated peak work rate from the 6MWT distance.

Participants will be monitored during training so that their HR is less than 85% of peak 6MWT HR, oxygen saturation (SpO_2_) is above 85%, and RPE is between 3 to 4 on the modified Borg RPE scale. If required, supplemental oxygen will be provided during exercise sessions to maintain SpO_2_ above 85%. Each exercise training session should take approximately 45 to 60 min to complete. Exercise training will be discontinued for participants who suffer any adverse events (including syncope, dizziness or marked worsening in oxygen desaturation during exercise) during exercise training. These participants will be referred to their physician (JF) for assessment and evaluation for suitability to continue the study.

Following the exercise training program, participants will be prescribed a standard home exercise training program. The home exercise program will consist of the participant being asked to walk 3–5 times per week for 20–30 min. Participants will be asked to walk at an RPE similar to which they achieved during the exercise training program. Compliance with the home exercise program will be evaluated at subsequent clinic visits.

### Control group

The control group participants will continue with their routine medical care and physical activity regimes for the 8-week study period without the additional exercise training intervention. During the 8-week period, participants allocated to the control group will receive a weekly phone call from the study coordinator to review any adverse events and to record exercise habits.

### Outcome measures

Baseline participant information including age, gender, anthropometrics (height and weight), co-morbid conditions and concomitant medications will be collected at the initial visit. In addition, various outcome measures will be undertaken at baseline, during and at completion of the 8-week study period. Clinical worsening will also be monitored for a minimum of 2 year period following commencement of the study (see below). All outcome measurements will be performed by a researcher blinded to the participants’ group allocation.

#### Primary outcome measure

The primary outcome measure will be exercise capacity assessed using the 6MWT at baseline and following the 8-week study period as per the American Thoracic Society and European Respiratory Society guidelines [22].

#### Secondary outcome measures

Cardiopulmonary exercise test is used to determine exercise capacity on a stationary cycle ergometer using previously described methods [23] before and after the 8-week study period. During the test, the participant will breathe through a mask so that oxygen uptake can be measured, using open circuit spirometry (Metamax, Cortex BXB, Lepzig, Germany) [24]. The metabolic system will be calibrated for volume and expired concentrations of oxygen and carbon dioxide prior to each test. Blood pressure, SpO_2_, HR and heart rhythm will be constantly monitored and the test will be supervised as per the ACSM guidelines [20]. All participants will commence cycling at 0 W for a 1 min period. The external workload will then be increased using a ramped protocol (5–10 W per minute, depending on disease severity and baseline exercise capacity), such that each test will be 8–12 min duration. At the completion of the test, participants will perform a supervised warm down. Peak minute average values for oxygen uptake ($\dot{\mathrm{V}}{\mathrm{O}}_2$), carbon dioxide production ($\dot{\mathrm{V}}{\mathrm{CO}}_2$), ventilation (${\dot{\mathrm{V}}}_{\mathrm{E}}$) and HR will be determined. In addition, breathing efficiency (${\dot{\mathrm{V}}}_{\mathrm{E}}/\dot{\mathrm{V}}{\mathrm{CO}}_2$), end-tidal CO_2_ (P_ET_CO_2_), and anaerobic threshold will be determined using previously described methods [25]. The same ramped protocol will be used both pre- and post-exercise training.

Health-related QoL will be measured using a disease-specific PH tool, the Cambridge Pulmonary Hypertension Outcome Review (CAMPHOR) [26, 27] and the 36 item short-form survey (22). Health-related QoL will be assessed at baseline and following the 8-week study period.

Cardiac function will be measured using both cardiac magnetic resonance imaging (CMRI) and echocardiography, with CMRI serving as the principal method. Left and right ventricular end-diastolic volume and end-systolic volume will be measured using CMRI (1.5 T Scanner, MAGNETOM Aera, Siemens Healthcare, Erlangen, Germany) from which stroke volume, ejection fraction, cardiac output and contractile reserve will be derived [28]. In addition, measures of both right and left ventricular mass will be obtained. These will be measured at rest and during 2 submaximal exercise intensities (at 30% and 60% of their respective peak workload determined from incremental exercise test) using the MRI cycle ergometer. During the CMRI, electrocardiogram (ECG) and SpO_2_ will be continuously monitored and the test will be supervised by a medical practitioner. Echocardiographic evaluation will be performed as part of standard clinical protocol on the same day of the 6MWT. This will be assessed both at baseline and following the 8-week study period. Echocardiographic images will be acquired using a 4 MHz matrix array cardiac transducer (Vivid 7, General Electric, Milwaukee, Wisconsin, USA). All images will be attained with the participant in a semi-recumbent position for both the apical and parasternal windows. Right ventricular systolic pressure will be estimated from the tricuspid regurgitation velocity as per standard guidelines [29]. Right ventricular systolic function will also be examined by assessing the tricuspid annular plane systolic excursion.

Adverse responses or events including syncope, dizziness or marked worsening in oxygen desaturation during exercise will be monitored and recorded for the duration of the 8-week exercise training period. Such adverse events will also be assessed with weekly phone calls and recorded for the participants in the control group during the 8-week study period.

Long term outcomes will be analysed by examining survival and time to clinical worsening during routine follow-up clinic visits for a 2-year period following the participant’s commencement of the study. We will base this measure on the recent PH intervention studies [30–32]. For the purpose of this study clinical worsening will be defined as [30]:

- PH-related death or
- listing for/completed lung transplantation or
- hospitalization for PH or clinical worsening leading to initiation of new PH-specific treatment or
- World Health Organisation (WHO) functional classification deterioration and a > 15% decrease in 6MWT from baseline.

Participants will be assessed for clinical worsening every 3–6 months following randomisation during routine clinical visits. A panel of two clinical experts, blinded to group allocation, will independently evaluate each participant for clinical worsening. Decisions will be moderated by a third expert, blinded to group allocation, in the event that the panel members reach a different decision.

### Data analysis

Individual data will be de-identified, coded and entered into a password protected, secure database. The group allocation for each individual participant will be stored in a separate file at the time of allocation. Data will be double entered and range checks made on values to ensure data integrity. On completion of the study, the primary investigator (PI) will have access to the final trial dataset. An intention-to-treat analysis will be used to avoid bias in estimating the treatment effect for this superiority study, with inclusion of all measures in all available participants, regardless of whether they completed the intervention. We will use a generalised linear mixed model approach which can accommodate missing data points often encountered in longitudinal datasets and provides an ideal way of dealing with missing values or dropouts [33]. Equivalent parametric and non-parametric tests will be conducted where data does not meet normative assumptions. Probability of a Type I error will be set at 0.05. Survival and clinical worsening (event-free survival) will be estimated by the Kaplan-Meier method, using a stratified log-rank test for group comparisons. The commencement of the exercise program or control period will be used as the index date for determining survival or clinical worsening. Participants who have no clinical worsening event will be included up to their first clinic visit after the 2-year period study commencement.

### Sample size and power calculations

A total of 50 participants will be recruited into this study. We have allowed for a 10% drop out over the 8-week period to ensure that 45 participants will complete the study. Where appropriate, sample sizes for the primary and secondary outcome measures have been determined using weighted effect sizes calculated from randomised controlled trials of exercise training in pulmonary hypertension [15].

For the primary outcome measure, i.e. change in the 6MWT distance, the study is powered at 0.91 using a 2-tailed test for independent samples and a weighted effect size of 1.01. For the peak exercise capacity ($\dot{\mathrm{V}}{\mathrm{O}}_{2\mathrm{peak}}$), this study is powered at 0.95 using an pooled, weighted effect size of 1.10 [15]. The primary measure for QoL in the current study will be the CAMPHOR. For the role physical domain in the CAMPHOR, the study is powered at 0.90 based on a weighted effect size of 0.99 [15].

The principle measure for changes in cardiac function will be made using CMRI. Recently Addetia and colleagues suggested that a per group sample size of 23 participants would detect a 5% change in right ventricular ejection fraction [34] measured using CMRI with a power of 80% and the probability of a Type I error of 0.05. The authors suggest that for a 10% change in right ventricular ejection fraction, a smaller sample size of 6 participants would be required. Based on a conservative estimate of a 5% change in right ventricular ejection fraction, the current study would have approximately 80% power to detect changes in right ventricular systolic function.

To determine the power of the current study to detect a change in clinical worsening, we have used data from previous non-RCT examining impact of exercise training on clinical worsening in PH [31] which reported the probability of being free from clinical worsening following an exercise intervention was 0.74. From REVEAL (Registry to Evaluate Early and Long-term Pulmonary Artery Hypertension Disease Management), the 2-year probability for a participant to be free from clinical worsening was 0.36 [35]. Combining these would give a risk ratio (RR) for clinical worsening following exercise training of 0.41 (i.e. = risk in exercise group 0.26/risk in control group 0.64). Based on this risk ratio, then a sample size of 45 would detect this RR with 30% power and α = 0.05 (two-tailed test). We have assumed a 5% loss to follow-up over a 2-year period. Whilst this appears conservative, individuals with PH have regular (every 3–6 months) clinic appointments to monitor progress and therapy. Although this study is underpowered to detect these long-term outcome measures, one may argue that including this outcome measure will provide important long-term pilot data for future studies.

### Data monitoring

A data safety monitoring board (DSMB) will be established to monitor the project. The DSMB will meet during the trial with the role to: monitor and review participant safety in the trial; monitor efficacy based on pre-planned interim data analyses (if required); and to review participant recruitment, accrual, retention, and withdrawal. The responsibility of the DSMB will be exercised by providing recommendations about continuing, modifying or stopping the trial. The DSMB will be advisory to the PI. A copy of the DSMB charter can be found in a secure file located on the hospital server along with all documents related to the ethical conduct of the project including the study protocol, informed consent and relevant questionnaires.

## Discussion

Despite advances in medical therapies, PH is associated with considerable morbidity and premature mortality. Patients with PH frequently report exertional dyspnoea and fatigue, functional limitation and impaired QoL. Exercise training is recognised as a valuable adjunct in the management of many chronic respiratory and cardiac conditions, with well-established benefits including improvements in exercise capacity and QoL [36, 37]. Whist a limited number of randomised, controlled exercise training studies have demonstrated similar benefits in stable PH patients, our understanding is limited with regard to the efficacy of a standard, outpatient-based exercise programs for PH, mechanisms of improvements, the potential effects on prognosis and the optimal exercise prescription parameters [38]. This has resulted in uncertainty amongst guideline developers and health care professionals in terms of recommending and prescribing exercise for this high risk patient population.

This study will be the largest randomised controlled trial to comprehensively examine the safety and efficacy of exercise training in an outpatient setting for medically stable PH patients and changes in cardiac function at rest and during exercise measured using CMRI during exercise. The specific aims of this study are to evaluate whether supervised exercise training leads to improvements in exercise capacity, cardiac function, QoL and long-term prognosis, without causing any significant training-related adverse effects. Hence, it will be the first study of its kind to evaluate the impact of exercise training on cardiac function and disease progression in the PH patient population. With the use of gold standard approaches, we will be able to determine the effect of exercise training on cardiac function during both rest and exercise, as well as whether changes in cardiac function accompany any changes in exercise capacity. As such, the results of this study will have important implications for clinical practice and future guideline recommendations.

## Abbreviations

$\dot{\mathrm{V}}{\mathrm{CO}}_2$: Carbon dioxide production
$$ \dot{\mathrm{V}}{\mathrm{O}}_2 $$: Oxygen uptake
$\dot{\mathrm{V}}{\mathrm{O}}_2\mathrm{peak}$: Peak exercise capacity
$$ {\dot{\mathrm{V}}}_{\mathrm{E}} $$: Ventilation
${\dot{\mathrm{V}}}_{\mathrm{E}}/\dot{\mathrm{V}}{\mathrm{CO}}_2$: Ventilatory equivalent for Carbon Dioxide
6MWT: Six minute walk test
CAMPHOR: Cambridge pulmonary hypertension outcome review
ECG: Electrocardiogram
HR: Heart rate
MRI: Magnetic resonance imaging
NYHA: New York Heart Association
P_ET_CO_2_: End tidal carbon dioxide
PH: Pulmonary hypertension
PI: Principal investigator
QoL: Quality of Life
RCT: Randomised controlled trial
REVEAL: Registry to evaluate early and long-term pulmonary artery hypertension disease management
RPE: Rating of perceived exertion
SpO_2_: Oxygen saturation
WHO: World Health Organisation
